# Epidemiology and control of trachoma in the state of Ceará, Northeast Brazil, 2007-2021

**DOI:** 10.1590/0037-8682-0207-2022

**Published:** 2023-01-23

**Authors:** Adjoane Maurício Silva Maciel, Alberto Novaes Ramos, Vivian da Silva Gomes, Anderson Fuentes Ferreira, Nádia Maria Girão Saraiva de Almeida, Daniela Vaz Ferreira Gómez, Joana da Felicidade Ribeiro Favacho, Manuella Maurício Silva Maciel, Antônio Lucas Delerino, Roberto da Justa Pires

**Affiliations:** 1 Universidade Federal do Ceará, Faculdade de Medicina, Programa de pós-graduação em Saúde Pública, Fortaleza, CE, Brasil.; 2 Secretária Municipal de Saúde, Russas, CE, Brasil.; 3 Universidade Federal do Ceará, Faculdade de Medicina, Departamento de Saúde Comunitária, Fortaleza, CE, Brasil.; 4 Universidade Estadual do Ceará, Mestrado Profissional em Saúde da Criança e do Adolescente, Fortaleza, CE, Brasil.; 5 Secretaria de Estado da Saúde, Fortaleza, CE, Brasil.; 6 Ministério da Saúde, Secretaria de Vigilância em Saúde, Brasília, DF, Brasil.; 7 Ministério da Saúde, Secretaria de Ciência, Tecnologia e Insumos Estratégico-SCTIE, Instituto Evandro Chagas, Belém, PA, Brasil.; 8 Universidade Federal do Ceará, Instituto de Cultura e Arte, Fortaleza, CE, Brasil.; 9 Universidade Federal do Ceará, Departamento de Enfermagem, Fortaleza, CE, Brasil.

**Keywords:** Trachoma, Epidemiology, Surveys, Ceará, Brazil

## Abstract

**Background:**

To analyze the epidemiology, surveillance, and control strategies for trachoma in the state of Ceará, northeast Brazil, from 2007 to 2021.

**Methods:**

This ecological study was based on secondary data from the Information System on Notifiable Diseases of the Secretary of Health of the state of Ceará. Data from school and home surveys for trachoma detection obtained during the study period were analyzed, the percentage of positivity was estimated, and sociodemographic and clinico-epidemiological factors were investigated.

**Results:**

The coverage of trachoma surveillance and control actions in Ceará municipalities increased from 12.5% in 2007 to 55.9% in 2019, but with an average restriction of 8.0% during the COVID-19 pandemic. The estimated trachoma positivity (mean overall positivity) was less than 5.0% (2.76%, 95% CI 1.2-5.2), with a higher proportion of cases in the 5-9-year age group (45.0%, 95% CI 44.6-45.4), in females (53.2%, 95% CI 52.8-53.6), and rural areas (52.6%, 95% CI 52.2-53.0). Positivity above 10.0% was observed in the Litoral Leste/Jaguaribe and Sertão Central regions, with a higher occurrence of the follicular inflammatory clinical form (98.1%, 95% CI 98.0-98.2).

**Conclusions:**

Trachoma remains in the state of Ceará and is likely underreported. Despite recent advances, the fragility of health surveillance activities compromises the recognition of the actual magnitude and distribution of trachoma in the state. Accurate information is fundamental for planning, monitoring, and evaluating surveillance and disease control.

## INTRODUCTION

Trachoma is chronic and relapsing keratoconjunctivitis caused by *Chlamydia trachomatis,* which remains the leading cause of blindness of infectious origin worldwide^1^. In addition, trachoma belongs to the group of neglected tropical diseases (NTDs)^2^ and is associated with poverty^1^ ^,^ ^3^, low education, and inadequate sanitary conditions^2^.

More severe cases are associated with a higher frequency of reinfection episodes, increased inflammatory reaction, and in turn, conjunctival scarring and necrosis. Consequently, eyelid traction (entropion), inversion of the eyelashes with corneal affection (trachomatous trichiasis), and ulcerations (corneal opacity) may result in constant pain, intense photophobia, reduced visual acuity, and blindness^4^.

Trachoma surveillance and control strategies include the development of timely diagnosis and treatment of cases and adopting relevant prevention and control measures based on regular surveys, active searches for cases, and home visits to contacts^4^.

To evaluate and monitor the performance of the Trachoma Surveillance and Control Program (in Portuguese: Programa de Vigilância e Controle do Tracoma [PVCT]), we considered an operational indicator that reflected the prevalence of the disease in a specific population. This operational indicator is commonly used to analyze the results of school surveys^5^.

The World Health Organization (WHO) has set the following targets for the elimination of trachoma as a public health problem: prevalence of follicular inflammatory trachoma (FT) <5.0% in children aged 1-9 years and prevalence of trachomatous trichiasis (TT) “*unknown to the health system*” less than 0.2%, both in previously endemic areas, with evidence of the health system ensuring the identification and management of incident cases of TT^6^. 

According to the WHO, TT, which is “*unknown to the health system*”, excludes TT in individuals with postsurgical recurrence, who have refused surgical treatment and/or have not undergone surgical intervention, but for whom surgery has already been scheduled^7^. 

Although the global elimination of trachoma has not been achieved, there has been a reduction in the number of at-risk individuals worldwide from 142.2 million in 2019 to 136.9 million in 2020. This demonstrates collaboration with the Sustainable Development Goals agenda for NTDs for 2021-2030 of “*ending the epidemics of AIDS, tuberculosis, malaria, and neglected tropical diseases, and combating hepatitis, waterborne, and other communicable diseases*”^1^.

In Brazil, the prevalence of active trachoma is under the limits recommended for eliminating the disease^8^. However, the sequelae form of the disease, the TT “*unknown to the health system*,” had an estimated prevalence of 0.22% in some areas, such as northwestern Ceará. This borders the critical value of 0.2% in non-indigenous areas but is within the confidence interval (0.06-0.44)^8^ revealed in the National Survey for the Validation of the Elimination of Trachoma as a Public Health Problem, developed by the Ministry of Health, for the period 2018-2019, for non-indigenous areas.

Despite its epidemiological relevance and the elimination validation process under development, the level of endemicity in some municipalities in the state of Ceará^9^ is unknown. Furthermore, prevalence is recognized as bordering the critical value for the sequelae of the disease^8^. There is a lack of research quantifying the current epidemiological situation in some areas of the state^10^.

Faced with the challenges presented and the need to consolidate information that will identify the epidemiological and operational indicators of trachoma control, this study aimed to analyze the epidemiological and operational scenarios of trachoma control in the state of Ceará, Northeast Region of Brazil, from 2007 to 2021. Therefore, a critical analysis of the occurrence and distribution of the disease according to the endemicity parameters was undertaken to provide a guide for planning, evaluation, and recommendations to strengthen surveillance and disease control.

## METHODS

**FIGURE 1:**
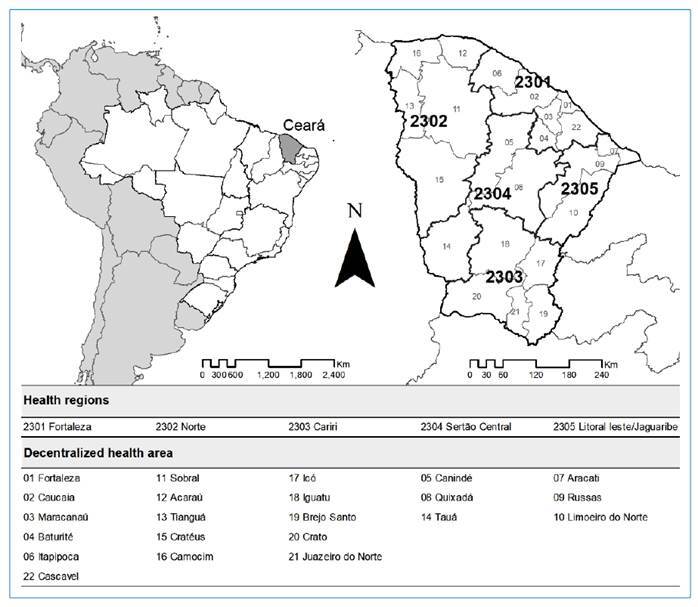
Health regions of the state of Ceará, Brazil.

We conducted an ecological study based on epidemiological data on trachoma in the state of Ceará (Figure 1), reported in the Information System on Notifiable Diseases (SINAN) by the Secretary of Health of the State of Ceará. This database contains the records of surveys conducted in municipalities in the state of Ceará. Information was collected from individuals who were examined and diagnosed with trachoma upon external eye examinations. Data from 2007 to 2021 were used in this study. Beginning in 2007, the results of trachoma investigations were made available in SINAN by the Secretary of Health of the state of Ceará. 

During the study period (2007-2021), school and household surveys were developed concomitantly. Between 2011 and 2013, household surveys predominated, with a proposed sampling of 80% of households. School surveys continued in the period 2013-2017 as part of the Ministry of Health’s “National Campaign on Leprosy, Worms, Trachoma, and Schistosomiasis”. During this period, external eye examinations were conducted among public school students aged 5-14. From 2017, following the "Panel of Strategic Health Surveillance Indicators of the State of Ceará”, the proposed sample was 50% or more of students aged 1-9 in the public elementary school network of the municipalities considered a priority that was part of each health region. The contents of schoolchildren with trachoma were examined through household surveys.

Our analysis was based on calculating absolute and relative frequencies using the percentage of positivity. To this end, we counted the number of positive cases of trachoma, divided it by the total number of individuals examined, and multiplied this by 100. The operational parameters proposed as a reference by the Brazilian Ministry of Health consider variations in endemicity levels, with values <5.0%, 5.0-9.9%, and ≥10.0%.

Demographic and clinic-epidemiological patterns were characterized according to the percentage of positivity for trachoma and represented based on the levels of endemicity in the period 2007-2021, according to the following variables: health regions of the state of Ceará (Figure 1), using the administrative management model adopted by the Health Department of the state of Ceará, with subdivisions and respective number of municipalities (Litoral Leste/Jaguaribe [20], Cariri [45], Sertão Central [20], Norte [55], and Fortaleza [44]); sex (male and female); age group (0-4, 5-9, 10-14, and ≥15 years); area of residence (urban, peri-urban or rural); clinical forms, according to the case definition for the disease based on the active forms (trachomatous inflammation follicular [TF], presence of ≥ 5 follicles > 0.5 mm in the upper tarsal conjunctiva of the eye; trachomatous inflammation intense [TI], inflammatory thickening of the upper tarsal conjunctiva, not allowing visualization of > 50% of the deep tarsal vessels); and sequelae forms (trachomatous scarring [TS], presence of scarring of the upper tarsal conjunctiva; trachomatous trichiasis [TT], presence of at least one of the lashes rubbing the eyeball or evidence of recent epilation of lashes on the upper eyelid; and corneal opacity [CO], opacity obscuring the pupillary margin)^4^.

The organization and management of the database were performed using the statistical program Stata 11.2 (Stata Statistical Software, StataCorp LP, College Station, TX, USA) through the construction of tables and graphs. 

The temporal patterns of positivity for the disease were analyzed considering the variation in endemicity levels in the health regions of the State of Ceará for the entire period of 2007-2021. The data were exported and joined to the shapefiles of the municipalities of the state of Ceará, according to the standards of the Brazilian Institute of Geography and Statistics, using the software QGIS version 2.18.6 (licensed under the General Public License [GNU], available at https://qgis.org/pt_BR/site/. We constructed thematic maps by verifying the spatial patterns of endemicity in the pre- coronavirus disease (COVID-19) pandemic period (2007-2010, 2011-2014, 2015-2019) and during the COVID-19 pandemic (2020-2021).

This study was approved by the Ethics Committee of the Hospital São José de Doenças Infecciosas (HSJ) of the State of Ceará (opinion number 5.132.182).

## RESULTS

**FIGURE 2:**
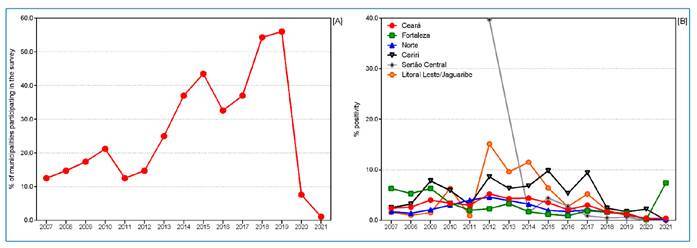
Municipalities participating in school and household surveys **[A]** and positivity for trachoma in health regions **[B]** Ceará State, Brazil, 2007-2021.

**FIGURE 3:**
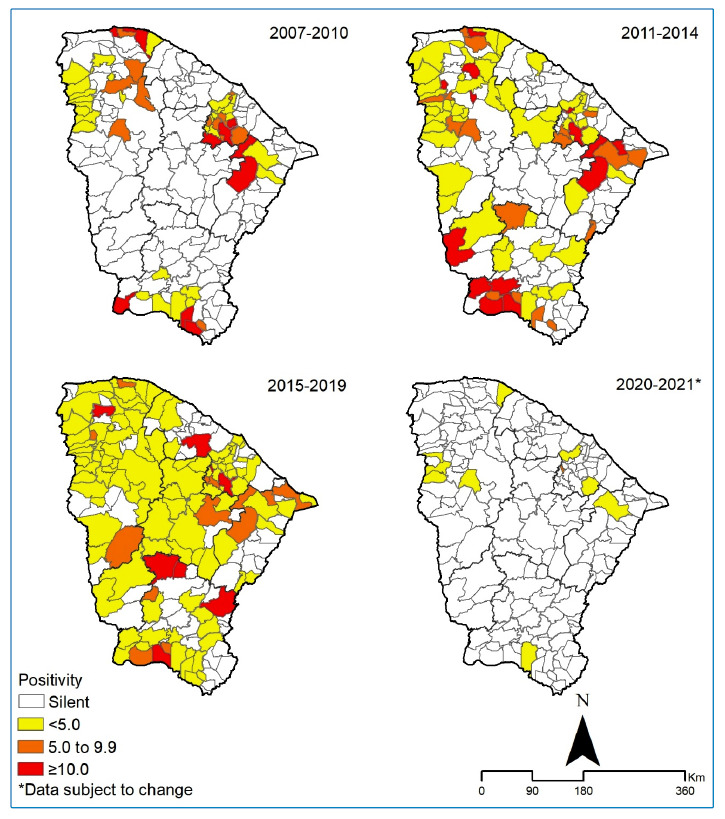
Trachoma endemicity in Ceará State, by municipality, Brazil, 2007-2021.

A total of 2,048,038 people underwent external eye examinations during school and household surveys in the state of Ceará between 2007 and 2021. Among them, 56,612 cases of trachoma were identified, with cases detected each year. The overall mean trachoma positivity was < 5.0% (2.76%, 95% CI 1.2-5.2%) (Figure 2B). In 2020 and 2021, during the COVID-19 pandemic, positivity remained < 5.0% (0.3-0.4%), however, a reduced number of municipalities (14 and 2, respectively) and schools (11,176 and 683, respectively) were analyzed (Figure 2A). There was no significant difference in annual trachoma positivity in the state of Ceará during the study period. However, exceptions were observed, with percentages of ≥ 10.0%, in the health regions Litoral Leste/Jaguaribe in 2012 and 2014 and in Sertão Central in 2012 (Figure 2B) (Figure 3).

The maintenance of endemicity of the disease in all surveyed areas was confirmed, with positivity of 5.0-9.9%, in Fortaleza (2007-2009), Cariri (2009-2010, 2012-2017), and Litoral Leste/Jaguaribe (2010, 2013, 2015, and 2017) (Figure 2B) (Figure 3).

In terms of the operational aspects of disease control, it is noteworthy that, from the total of 184 municipalities, a small number of municipalities had developed mechanisms for surveillance and control of trachoma at the beginning of the study period (2007-2010; 12.5%, 23/184), which increased between 2011-2014 (36.9%, 68/184) and then again between 2015-2019 (55.9%, 103/184) (Figure 3). Further, the significant participation of the North health region, with 46% (942,582/2,048,038) of the total number of people examined in the state (2007-2021), should also be noted. Despite this, many municipalities had not developed surveys or notifications of cases (44%, 81/184) by the end of 2019 (Figure 2A). Further, in the last biennium of the study (2020-2021), and as a result of the COVID-19 pandemic, such activities became quite restricted, with < 8% (11,859) of the average number of tests performed during the study period (Figure 2A). Regarding positivity, we note an increase in the 2007-2012 time period (+1.17%) and a decrease in the 2012-2021 period (-10.9%).

**FIGURE 4:**
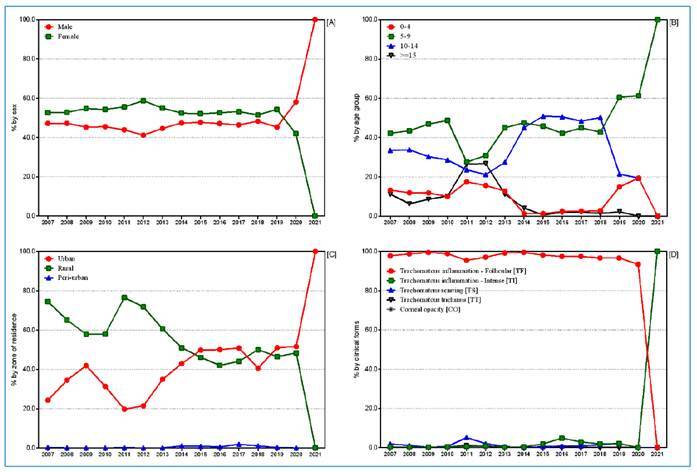
Percentage of positivity for trachoma according to sex **[A]** age group **[B]** area of residence **[C]** and clinical form **[D]** in health regions, Ceará State, Brazil, 2007-2021.

In the context of demographics, we found 91.7% (95% CI 91.5-92.0) of cases in the 0-14-year age group and 7.8% (95%CI 7.6-8.1) of cases in the ≥15-year age group (Figure 4B). No age group classification was recorded in 0.5% (247) of cases. There was an overall predominance in the 5-9-year age group (45.0%, 95%CI 44.6-45.4), followed by the 10-14-year age group (40.8%, 95%CI 40.4-41.2). Interestingly, an inverse result was observed between 2015-2018, where a higher proportion of positivity was obtained in the 10-14-year age group (Figures 4A,B).

Results showed a greater occurrence of trachoma among females (53.2%, 95% CI 52.8-53.6) in all years analyzed, except for 2020 and 2021. 

The overall occurrence of the disease was more evident among those living in rural areas of the state of Ceará (52.6%, 95% CI 52.2-53.0), particularly in the first half of the study period. However, a higher prevalence in urban areas was observed for some years (Figure 4C).

Among the active clinical forms, TF was predominant (98.1%; 95% CI 98.0-98.2), varying between 93.5% and 99.5% of the total number of cases throughout the historical series. Although the search was directed toward the active forms of the disease, in school surveys, a low proportion of sequelae was detected among the people examined (Figure 4D) (Figure 3).

## DISCUSSION

This study demonstrated a persistent pattern of trachoma endemicity in the state of Ceará, which was similar to that estimated at the national level^11^. Further, we observed a progressive reduction in the occurrence of the disease and an average positivity below the recommended level for the elimination of trachoma as a public health problem. 

The improvement in access to water for human consumption through the implementation of cisterns for low-income rural populations, the increase in coverage of primary healthcare (PHC), and the continuity and strengthening of surveillance and control mechanisms of the disease are national strategies^8^ that favor improvement of living conditions and, consequently, the percentage of the percentage of positivity below 5.0% among municipalities that conduct surveys. According to WHO’s recommendations, this epidemiological situation is favorable for reaching the goal of eliminating trachoma as a public health problem^8^.

However, we emphasize the presence of different endemicity patterns and maintenance of higher positivity in specific areas, in the period 2007-2019 reinforcing the need to develop population-based studies to estimate the real magnitude of disease prevalence.

From an operational point of view, we verified that there is still an insufficient expansion of PVCT coverage in the state of Ceará. In addition, a considerable percentage of municipalities did not actively diagnose, notify, or treat cases of trachoma. Thus, there is still a lack of awareness of the level of endemicity of the disease among some municipalities in the state of Ceará.

To strengthen the response to trachoma and other diseases in which the results of national programs were considered insufficient and incompatible with the capacity of the Unified Health System (SUS) to solve the health problems of the population, the Ministry of Health published the “*Integrated Plan of Strategic Actions for the Elimination of Leprosy, Filariasis, Schistosomiasis and Onchocerciasis as a public health problem, trachoma as a cause of blindness and control of geohelminthiases 2011-2015*” in 2012, and listed 267 municipalities that were to be considered a priority to receive financial resources for the strengthening of disease surveillance and control^5^.

To support the strengthening of these measures, in addition to enabling the optimization of resources in the fight against this group of diseases, the “*National Campaign of Leprosy, Verminosis, Trachoma, and Schistosomiasis*” was implemented in five editions, from 2013 to 2018, among public school students aged 5-14 years^12^. This included the use of an active search strategy and timely treatment of at least 80% of cases and the development of promotional and preventive actions for the health of this population^4^.

In the state of Ceará, 46 municipalities were considered priorities due to the high burden of disease; low coverage of public services of water, sewage, and rubbish; low human development index; and the high percentage of the population in poverty conditions, according to the United Nations Development Program^4^. As a result of this initiative, there was a growing involvement of municipalities and an expansion of the coverage of trachoma surveillance and control in the state of Ceará. Instituted in the context of health surveillance, PHC, and the School Health Program, these campaigns were an efficient strategy to access the most vulnerable populations^13^ through health education activities, active search, diagnosis, treatment, referral of cases to a specialized network, and the control and recording of cases in SINAN and Form-SUS^12^. The intervention results were also enhanced by the opportunity of reaching a large number of schoolchildren, which was possible due to the inclusion of schools in the initiative and the presence of both children and adolescents in this environment^12^.

Associated with these strategic and operational actions, capacity building (number of actions=13) was conducted in surveillance and trachoma control activities with theoretical and practical approaches in the capital and interior of the state of Ceará. This was done through the National Policy of Continuing Education in Health to improve the work processes of professionals (number of actions=135) in the areas of health surveillance and PHC^14^.

Although the coverage of the PVCT was limited during the COVID-19 pandemic, activities to eliminate the disease were maintained, even under the COVID-19 restrictions, and while awaiting the return of school surveys which would come with the return of a functioning public school network and epidemiological and operational conditions of the municipalities.

Pandemics constitute a challenge not only for global health but also for implementing public health policies to control and eliminate^15^ NTDs. The main challenges are the limitation of community work, the reduced availability of health professionals, and the lack of funding for control activities^15^. In this context, maintaining activities to promote eye health during the COVID-19 pandemic is critical; however, it is also possible to implement precautionary measures^16^ and risk-benefit analyses to provide health services and progress toward eliminating trachoma as a public health problem^17^. 

To understand the current epidemiological situation of the disease in the country and to guide the national planning of trachoma surveillance and control activities^18^, the Ministry of Health developed, between 2018-2019, the first stage of the National Survey for the Validation of the Elimination of Trachoma as a public health problem in non-indigenous areas at epidemiological and social risk^8^.

In addition, it proposed guidelines for surveillance and control of the disease through strategic actions: 1) active search activities in hyperendemic communities (≥10%) and health education activities, in conjunction with PHC and the educational sector; 2) treatment of inflammatory cases and external eye examination for household and institutional contacts; 3) training for health professionals; 4) exclusive use of SINAN with regular monitoring of data and supervision at diagnosis of trachoma cases in municipalities with prevalence ≥ 5%; 5) verification of the occurrence of TT in populations aged ≥ 15 years, particularly those > 50 years, in areas considered old pockets of the disease; 6) sensitization of specialized care for identification of TT cases, referral, registration, and documentation of surgical interventions to validate the elimination of trachoma as a public health problem; and 7) encouragement of coordination with the SUS Management Councils to support the development of actions, as well as other programs of NTDs aimed at resource optimization and operational logistics in endemic areas^6^.

The sociodemographic characteristics of people with trachoma in the state of Ceará are similar to those in other states in Brazil^19^ ^,^ ^20^ as well as in other countries^21^, with a higher frequency of trachomatous infection among children aged 5-9 years, indicating the main reservoir of infection^4^, and in females, which is probably related to more effective behavior through direct contact^19^, an important form of transmission of infection. However, studies on schoolchildren in Brazil have not characterized cases of trachoma among children in the 1 to 4-year-old age frame because this population is not enrolled in public schools^20^.

Demographic data also indicated a higher concentration of trachoma cases in rural territories in the state of Ceará. This is likely a result of the greater difficulty in accessing health services in rural areas due to factors such as geographic distance and transportation costs^22^. In this sense, they negatively influence the detection, treatment, and control of cases^23^, in addition to sanitary conditions, even with improved access, increasing visual impairment in rural populations^19^. 

Clinical evaluation showed a predominance of TF among children under 15 years of age, which has also been reported in studies on schoolchildren in other regions in Brazil^24^ and other countries^21^. Although the frequency of TS, TT, and CO forms increased in a directly proportional manner with age, the occurrence of sequelae among schoolchildren in the state demonstrates the need to supervise record-keeping and diagnoses of trachoma cases^25^. This highlights the importance of activities for treating and controlling cases and contacts of trachoma^23^.

It is recommended that the occurrence of sequelae be investigated, especially TT and entropion, with the expansion of access to surgical services for the correction of these sequelae^11^ and studies to better understand the evolution of severity and evidence of association with the active form of the disease^26^.

The reduced number of adult/elderly sequelae in this study was due to the primary use of schoolchildren targeted surveys, with home-based examinations and treatment for contacts of trachoma cases.

The amplification of effective interventions by the global trachoma community to the components of facial hygiene and environmental improvement through the SAFE strategy (*surgery, antibiotics, face washing, environmental health*) and evaluation and monitoring of control actions are also necessary to reduce visual impairment and/or trachomatous blindness^27^. 

The study has some limitations, including restricted information due to “silent” municipalities, the absence of compulsory case notification, and the incomplete recording of variables in the database. Further, the characterization of data exclusively from positive cases limits the epidemiological analysis of the situation. The notification of cases represents an important tool for evaluating epidemiological situations through the recording and monitoring of data to strengthen the surveillance and control of diseases and illnesses^5^.

Another important aspect is the limitation of the information required to calculate the percentage of positive results for the disease. Improvements in data sources, integrated information systems, completeness of data recording and evaluation, monitoring, and capacity for analysis and use of data at all levels are challenges faced in planning and decision-making at the local and national levels for NTD programs^28^.

Despite the insertion of the indicator “Proportion of schoolchildren examined for trachoma” in the Health Surveillance Strategic Indicators Panel of Ceará (2018), there remains a need for the application of standard methods for the development of positivity surveys and the proper use of this indicator for the evaluation and monitoring of trachoma surveillance and control actions, in addition to implementing the registration of demographic and clinical data of people por examined for trachoma and their contacts.

Although prevalence levels are below the threshold for the elimination of the disease as a public health problem^4^, this study reiterates that trachoma is endemic in the state of Ceará, has limited PVCT coverage, probable under-recording, and a lack of knowledge regarding the levels of endemicity in some municipalities. The fragility of surveillance actions in health compromises recognition of the actual magnitude and distribution in the state. Accurate information is essential for planning, monitoring, and evaluating the surveillance and control of the disease, considering the characteristics of rural regions and specific populations, such as schoolchildren.

Implementation of guidelines for surveillance and control of the disease requires continued investments. This, together with additional research to strengthen surveillance capacity, represents essential elements to achieve and maintain the elimination of trachoma as a public health problem^29^.
